# Ischemia episode detection in ECG using kernel density estimation, support vector machine and feature selection

**DOI:** 10.1186/1475-925X-11-30

**Published:** 2012-06-15

**Authors:** Jinho Park, Witold Pedrycz, Moongu Jeon

**Affiliations:** 1 School of Information and Communications, Gwangju Institute of Science and Technology 1, , Oryong-dong, Buk-gu, Gwangju, Republic of Korea; 2 Department of Electrical and Computer Engineering, University of Alberta, Canada and Systems Research Institute, Polish Academy of Sciences, Warsaw, Poland

**Keywords:** Myocardial ischemia, Discrete wavelet transform, Kernel density estimation, Support vector machine, QRS complex detection, ECG baseline wandering removal

## Abstract

**Background:**

Myocardial ischemia can be developed into more serious diseases. Early Detection of the ischemic syndrome in electrocardiogram (ECG) more accurately and automatically can prevent it from developing into a catastrophic disease. To this end, we propose a new method, which employs wavelets and simple feature selection.

**Methods:**

For training and testing, the European ST-T database is used, which is comprised of 367 ischemic ST episodes in 90 records. We first remove baseline wandering, and detect time positions of QRS complexes by a method based on the discrete wavelet transform. Next, for each heart beat, we extract three features which can be used for differentiating ST episodes from normal: 1) the area between QRS offset and T-peak points, 2) the normalized and signed sum from QRS offset to effective zero voltage point, and 3) the slope from QRS onset to offset point. We average the feature values for successive five beats to reduce effects of outliers. Finally we apply classifiers to those features.

**Results:**

We evaluated the algorithm by kernel density estimation (KDE) and support vector machine (SVM) methods. Sensitivity and specificity for KDE were 0.939 and 0.912, respectively. The KDE classifier detects 349 ischemic ST episodes out of total 367 ST episodes. Sensitivity and specificity of SVM were 0.941 and 0.923, respectively. The SVM classifier detects 355 ischemic ST episodes.

**Conclusions:**

We proposed a new method for detecting ischemia in ECG. It contains signal processing techniques of removing baseline wandering and detecting time positions of QRS complexes by discrete wavelet transform, and feature extraction from morphology of ECG waveforms explicitly. It was shown that the number of selected features were sufficient to discriminate ischemic ST episodes from the normal ones. We also showed how the proposed KDE classifier can automatically select kernel bandwidths, meaning that the algorithm does not require any numerical values of the parameters to be supplied in advance. In the case of the SVM classifier, one has to select a single parameter.

## Background

Coronary artery disease is one of the leading causes of death in modern world. This disease mainly results from atherosclerosis and thrombosis, and it manifests itself as coronary ischemic syndrome [1].

**Figure 1:**
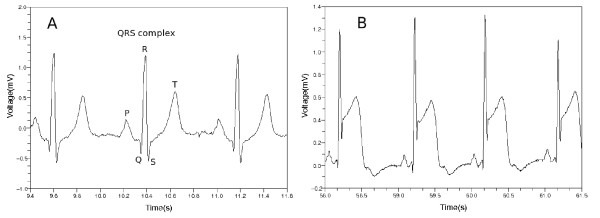
**Normal ECG and ST segment elevation.** (**a**) Normal ECG is divided into P, Q, R, S and T parts. The Q, R and S parts are called QRS complex in total. (**b**) This ECG waveform shows ST segment elevation.

When a patient experiences coronary ischemic syndrome, his or her electrocardiogram (ECG) shows some peculiar appearances. Each segment of ECG can be divided into P, Q, R, S and T waves as shown in Figure 1 where QRS complex and T wave represent ventricular depolarization and repolarization, respectively. In most cases of normal ECG, the ST segment has the same electric potential as the PR segment. When myocardial ischemia is present, however, the electric potential of the ST segment is elevated or depressed with respect to the potential of the PR segment [1,2]. When ischemia occurs, the PR segment is altered, or the ST segment deviates from normal level. If the PR segment moved instead of the ST segment, this looks as if the ST segment itself were modified. This is because the PR segment provides a kind of reference voltage level [1].

**Figure 2:**
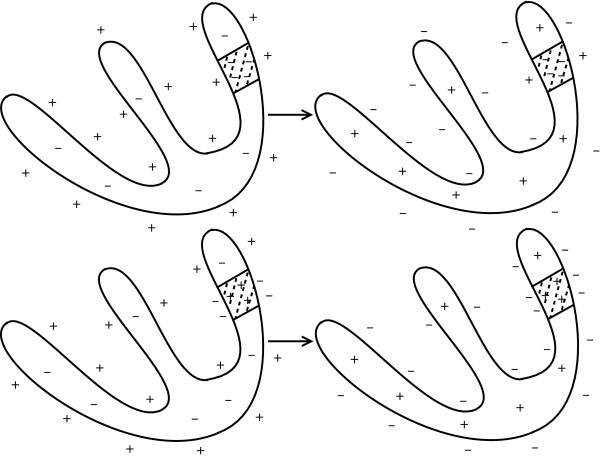
**Cause of ST segment deviation [**[1]**].** Left column shows distribution of electric charges before the ventricles contracts. The right column shows the charge distribution after the ventricles contracted. Shaded area represents that the area was affected by ischemia.

The ST segment deviation is mainly due to injury current in myocardial cells [1]. If the coronary artery becomes blocked by blood clot, some myocytes are affected to be unresponsive to depolarization, or to repolarize earlier than adjacent myocytes. In this case, voltage gradient can occur in the myocytes, and this comes to appear as ST-segment deviation in ECG [1]. Figure 2 shows two cases when the voltage level of the ST segment deviates from its normal position. The left column of the figure shows the distribution of electric charges around myocytes when the heart is in resting state. This is related to the PR segment in ECG. The right column shows the distribution of electric charges right after the ventricles contracted. This is related to the QRS complex and the ST segment in ECG. The shaded region represents the area being affected by myocardial ischemia. In the case of the upper row in Figure 2, there is no voltage gradient at first. After the ventricles contracted, however, the voltage gradient comes to appear because the injured area did not respond to electric depolarization. In the second case of the bottom row, there is no voltage gradient right after the ventricles contracted. In the left figure, however, there was initial voltage gradient, and this makes the PR segment to be modified. The PR segment acts as a reference voltage level when we judge whether the ST segment deviated from normal position. The modified PR segment makes us conclude that there was a ST segment deviation [1].

There are several approaches to detect ischemic ST deviations. Some researchers used the entropy. Rabbani et al. used the fact that signal perturbation of normal people is lower than the perturbation of ischemic patients. They computed entropy measure of wavelet subband of ECG signal, and classified the ECG by examining which signal exhibited a more chaotic perturbation [3]. Lemire et al. calculated signal entropy at various frequency levels. They computed the entropy in each wavelet scale [4]. Some used adaptive neuro-fuzzy inference system. Pang et al. used Karhunen-Loève transform to extract several feature values. They classified ECG signal by an adaptive neuro-fuzzy inference system [5]. Tonekabonipour et al. used multi-layer perceptron and radial basis function to detect ischemic episode. They classified ECG signals by adaptive neuro-fuzzy network [6]. There are many papers which used artificial neural network. Stamkopoulos et al. used nonlinear principal component analysis to analyze complex data. They classified ECG signal by radial basis function neural network [7]. Maglaveras et al. used neural network optimized with a backpropagation algorithm [8]. Afsar et al. used Karhunen-Loève transform to find feature values, and classified an input ECG by using a neural network [9]. Papaloukas et al. used artificial neural network which was trained by Bayesian regularization method [10]. There are papers studied some other approaches. Bulusu et al. determined morphological features of ECG, and classified the ECG data by support vector machine. Andreao et al. used hidden Markov models to analyze ECG segments. They detected ischemia episode by using median filter and linear interpolation [11]. Faganeli and Jager tried to distinguish ischemic ST episode and non-ischemic ST episode caused by heart rate change. To this end, they computed heart rate values, Mahanalobis distance of Karhunen-Loève transform coefficients and Legendre orthonormal polynomial coefficients [12]. Exarchos et al. used decision tree. They formed decision rules comprising specific thresholds, and developed a fuzzy model to classify ischemic ECG signals [13]. Garcia et al. considered root mean square of difference between the input signal and the average signal composed of first 100 beats. They adopted an adaptive amplitude threshold to classify ECG signal [14]. Murugan and Radhakrishnan used ant-miner algorithm to detect ischemic ECG beats. They calculated several feature values such as ST segment deviation from input ECG signal [15]. Bakhshipour et al. analyzed coefficients resulted from wavelet transform. They examined the relative quotient of the coefficients at each decomposition level of the wavelet transform [16].

We approached this problem by extracting feature values from a ECG waveform. We first found time positions of QRS complexes, and then determined values of the three features. We calculated the feature values for each heart beat, and averaged their values in five successive beats. After that, we classified them by the methods of kernel density estimation and support vector machine.

We showed techniques of removing baseline wandering and detecting time positions of QRS complexes by discrete wavelet transform. With these explicit methods of dealing with ECG, we could discriminate ischemic ST episode from normal ECG. We did not adopt implicit methods such as artificial neural networks or decision trees, because we considered it was important to utilize explicit features for processes of decision making. The artificial neural network has a kind of black box nature in its hidden layers [17], and a decision tree is apt to include several numerical thresholds [13].

## Methods

### Materials

We used the European ST-T database from Physionet. European ST-T database has 90 records which are two-channel and each two hours in duration [18,19]. Each record in this database has a different number of ST episodes. Overall there are 367 ischemic ST episodes in the database. Sampling frequency of each ECG data is 250 Hz.

We excluded 5 records because these had some problems. The records e0133, e0155, e0509, and e0611 had no ischemic ST episodes. The record e0163 had so limited ST episode whose length was just 31 seconds.

### Removing baseline wandering

The ST segments in ECG can be strongly affected by baseline wandering [20]. Main causes of the baseline wandering are respiration and electrode impedance change due to perspiration [20,21]. The frequency content of the baseline wandering is usually in a range below 0.5 Hz [20,21].

**Figure 3:**
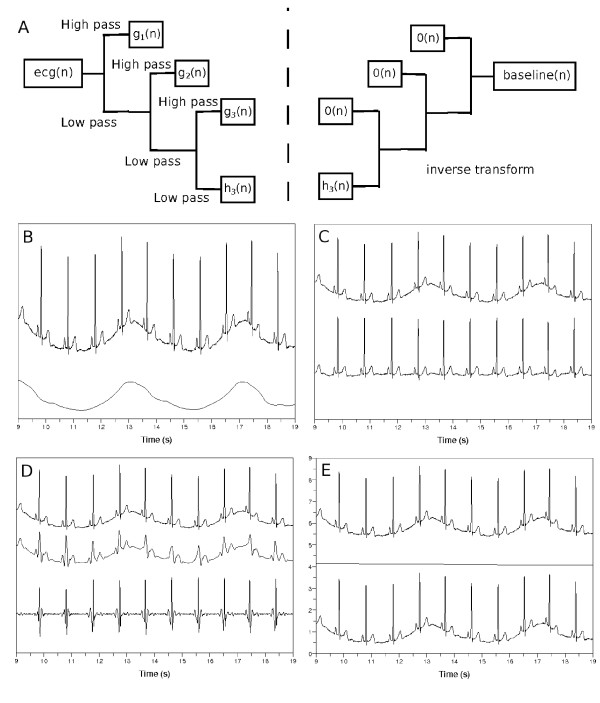
**Removing baseline wandering in ECG.** (**a**) Discrete wavelet transform of *ecg*(*n*) to find coefficient sequences *h*_*k*_(*n*),*g*_*k*_(*n*),*g*_*k*−1_(*n*),· · ·,*g*_1_(*n*). The $\vec{0}(n)$ means zero sequence. (**b**) Top: input ECG, *ecg*(*n*), bottom: wandering baseline in ECG, *baseline*(*n*). (**c**) Top: *ecg*(*n*), bottom: *ecg*(*n*)-*baseline*(*n*). When *k* is (**d**) too small or (**e**) too large, top: *ecg*(*n*), middle: *baseline*(*n*), bottom: *ecg*(*n*)-*baseline*(*n*).

We use discrete wavelet transform to remove baseline wandering in ECG. We transform signal vector into two sequences of coefficients, approximation and detail coefficients sequences [22]. We do this in each step in an iterative fashion, until we get an input signal whose length is smaller than the length of the filter which characterizes the wavelet. In our case, we used Daubechies8 wavelet with filter length of 8. The resulting approximation coefficient sequence becomes the input signal to the next discrete wavelet transform as shown in Figure 3(a) [22].

In each step, the coefficient sequence implies a band of frequencies. If the sampling frequency of a discrete ECG signal *ecg*(*n*) is *x*, we can determine a continuous and band-limited signal within frequency limits of $\left[0,\frac{x}{2}\right]$ by Nyquist sampling theorem [23]. Therefore if we have transformed the input signal *ecg*(*n*) into the approximation coefficient sequence *h*_1_(*n*) and detail coefficient sequence *g*_1_(*n*), then the frequency content of *g*_1_(*n*) is from $\frac{x}{4}$ to $\frac{x}{2}$, and the frequency content of *h*_1_(*n*) is below $\frac{x}{4}$. In this regard, if we have transformed the *ecg*(*n*) into the approximation coefficient sequence *h*_*k*_(*n*), and the detail coefficient sequences *g*_*k*_(*n*),*g*_*k*−1_(*n*),· · ·,*g*_1_(*n*), the frequency contents of *g*_*k*_(*n*),*g*_*k*−1_(*n*),· · ·,*g*_1_(*n*) become $\left[\frac{x}{2^{k+1}},\frac{x}{2^{k}}\right],\left[\frac{x}{2^{k}},\frac{x}{2^{k-1}}\right],\cdot \cdot \cdot \;,\left[\frac{x}{2^{2}},\frac{x}{2}\right]$ respectively [24,25].

To remove baseline wandering, we should choose appropriate wavelet scale. We follow argument similar to that presented by Arvinti et al. except that they used stationary wavelet transform instead of its discrete counterpart [26]. We remove signal components whose frequency content is less than 1/2 Hz [20,21]. If we have transformed the ECG signal *ecg*(*n*) into coefficient sequences *h*_*k*_(*n*),*g*_*k*_(*n*),*g*_*k*−1_(*n*),· · ·,*g*_1_(*n*), the frequency contents of *h*_*k*_(*n*) and *g*_*k*_(*n*) become $\left[0,\frac{x}{2^{k+1}}\right]$ and $\left[\frac{x}{2^{k+1}},\frac{x}{2^{k}}\right]$ respectively, where *x* is the sampling frequency. If we choose *k* as $\frac{x}{2^{k+1}}\leq \frac{1}{2}$$k=\left\lceil \underset{2}{\log}x\right\rceil$, the frequency content of the approximation coefficient sequence *h*_*k*_(*n*) becomes less than 1/2 Hz. Thus, we assign zero sequence $\vec{0}(n)$ to all the detail coefficient sequences *g*_*k*_(*n*),*g*_*k*−1_(*n*),· · ·,*g*_1_(*n*), and calculate inverse transform of $h_{k}(n),\vec{0}(n),\vec{0}(n),\cdot \cdot \cdot \;,\vec{0}(n)$ to form the *baseline*(*n*) in the bottom of Figure 3(b). If we subtract *baseline*(*n*) from *ecg*(*n*), we obtain the flattened signal like the one shown in Figure 3(c).

If we select a wrong wavelet scale *k* to find coefficient sequences of *ecg*(*n*), we obtain disappointing results. The flattened signal in Figure 3(c) is obtained when *k* is $\left\lceil \underset{2}{\log}250\right\rceil =8$, where 250 is the sampling frequency expressed in Hz. When select *k*=4 to use *h*_4_(*n*),*g*_4_(*n*),*g*_3_(*n*),*g*_2_(*n*),*g*_1_(*n*), we obtain a plot in Figure 3(d). The middle waveform, *baseline*(*n*), resulted from the inverse discrete wavelet transform of $h_{4}(n),\vec{0}(n),\vec{0}(n),\vec{0}(n)\vec{0}(n)$. This middle waveform is too detailed, so the bottom waveform *ecg*(*n*)-*baseline*(*n*) was negatively affected. When we select *k*=12, see Figure 3(e), the bottom waveform was not different from the input waveform *ecg*(*n*).

We adopt a discrete wavelet transform to retain the details of the ECG waveform because filtering by some cut-off frequency can deteriorate the quality of the ECG waveforms [27].

### Detecting QRS complexes

We have to select an appropriate wavelet scale to capture proper time positions of QRS complexes. We will deal with only the flattened ECG waveform *ecg*(*n*)-*baseline*(*n*) referred in the previous section. We will denote it as *fecg*(*n*).

First, we determine the sequences of wavelet coefficients of the *fecg*(*n*), obtaining *h*_*k*_(*n*),*g*_*k*_(*n*),*g*_*k*−1_(*n*),· · ·,*g*_1_(*n*) where $k=\left\lceil \underset{2}{\log}x\right\rceil$, *x* is sampling frequency. We assign zero to all the coefficient sequences except one, *g*_*j*_(*n*). Then, we calculate inverse transform of $\vec{0}(n)$ (approximation coefficients), $\vec{0}(n)$ (detail coefficients, onward), $\cdot \cdot \cdot \;,\vec{0}(n),g_{j}(n),\vec{0}(n),\cdot \cdot \cdot \;,\vec{0}(n)$ to obtain *pulse*(*n*). To find a protruding segment, that is, a QRS complex, we compute the score for each wavelet scale *j*,

(1) $$\mathrm{scor}e_{j}=\left|\underset{l}{\sum }\mathrm{fecg}(l)\frac{\left|\mathrm{pulse}(l)\right|}{\underset{m}{\sum }\left|\mathrm{pulse}(m)\right|}\right|.$$

**Figure 4:**
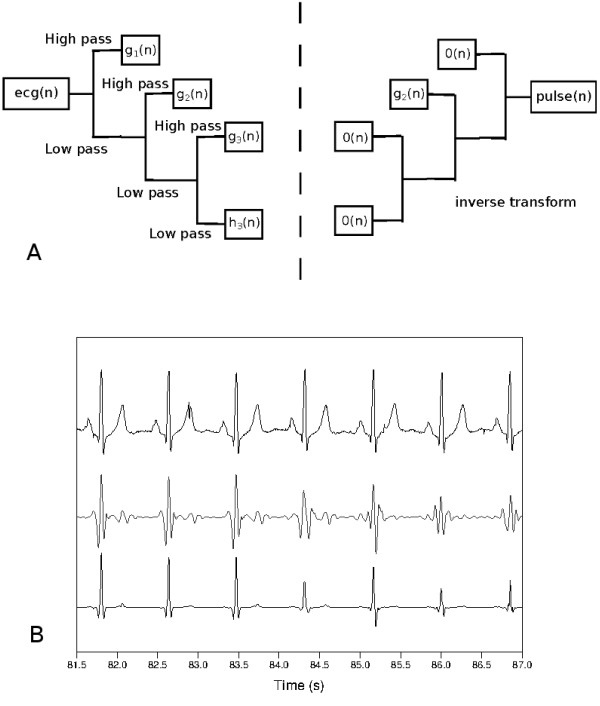
**Selection of wavelet scale to find the time positions of QRS complexes.** (**a**) Discrete wavelet transform and inverse transform. (**b**) Top: A flattened ECG waveform, *fecg*(*n*). Middle: waveform resulted from the inverse transform, *pulse*(*n*). Bottom: *fecg*(*n*)$\left|\mathrm{pulse}(n)\right|$.

We select the wavelet scale *j* which produces the largest drop of *score*_*j*_−*score*_*j* + 1_(*j*≥2). The bottom waveform in Figure 4(b) shows the time positions of QRS complexes when selecting this suitable wavelet scale.

After finding the locations of QRS complexes, we choose QRS onset and offset points in each QRS complex. We search QRS onset point in backward direction from a peak point in each QRS complex. We take the QRS onset point if the point is at the place of changing direction of rising and falling of *fecg*(*n*) twice. In the same way, we take the QRS offset point in forward direction from the peak point.

Algorithm 1 shows a process of removing baseline wandering and detecting QRS complexes.

#### 

##### Algorithm 1

A procedure to find time positions of QRS onset, peak and offset points. This procedure includes the method of removing baseline wandering in ECG. *nBeats* stands for the number of QRS peaks. It is the length of the sequences idx_QRS_Onset(*n*), idx_QRS_Peak(*n*) and idx_QRS_Offset(*n*). **Input:** Sampling_Hz, *ecg*(*n*)**Output:** idx_QRS_Onset(*n*), idx_QRS_Peak(*n*), idx_QRS_Offset(*n*)*k*$\left\lceil \underset{2}{\log}\text{Sampling\_Hz}\right\rceil$ Discrete wavelet transform (DWT) of *ecg*(*n*) into *h*_*k*_(*n*),*g*_*k*_(*n*),*g*_*k*−1_(*n*),· · ·,*g*_1_(*n*)**for***i*=1 to *k***do***g*_*i*_(*n*)$\vec{0}(n)$ {//$\vec{0}(n)$ means zero sequence.} **end for** Inverse wavelet transform (IDWT) of *h*_*k*_(*n*),*g*_*k*_(*n*),*g*_*k*−1_(*n*),· · ·,*g*_1_(*n*) into *baseline*(*n*)fecg(n)←ecg(n)−baseline(n) DWT of *fecg*(*n*) into *h*_*k*_(*n*),*g*_*k*_(*n*),*g*_*k*−1_(*n*),· · ·,*g*_1_(*n*)*h*_*k*_(*n*)>$\vec{0}(n)$*g*_*k*_(*n*)$\vec{0}(n)$**for***i*=1 to *k*−1**do**

$g_{i}^{\prime \prime }$(*n*)*g*_*i*_(*n*)

*g*_*i*_(*n*)$\vec{0}(n)$

**end forfor***i*=1 to *k*−1**do**

*g*_*i*_(*n*)$g_{i}^{\prime \prime }$(*n*)

IDWT of *h*_*k*_(*n*),*g*_*k*_(*n*),*g*_*k*−1_(*n*),· · ·,*g*_1_(*n*) into *pulse*(*n*)

$\mathrm{scor}e_{i}\leftarrow \left|\underset{l}{\sum }\mathrm{fecg}(l)\frac{\left|\mathrm{pulse}(l)\right|}{\underset{m}{\sum }\left|\mathrm{pulse}(m)\right|}\right|$

*g*_*i*_(*n*)$\vec{0}(n)$

**end for** chosen_scale ${\text{argmax}}_{2\leq i\leq k-2}\left\{\mathrm{scor}e_{i}-\mathrm{scor}e_{i+1}\right\}$$g_{\text{chosen\_scale}}(n)$$g_{\text{chosen\_scale}}^{\prime \prime }(n)$ IDWT of *h*_*k*_(*n*),*g*_*k*_(*n*),*g*_*k*−1_(*n*),· · ·,*g*_1_(*n*) into *pulse*(*n*)$\mathrm{needle}(n)\leftarrow \left|\mathrm{fecg}(n)\mathrm{pulse}(n)\right|$ Make idx_QRS_Peak(*n*) by searching for local maxima of *needle*(*n*)**for***i*=1 to *nBeats***doif**$\mathrm{fecg}\left(\text{idx\_QRS\_Peak}(i)\right)>0$**then**

j←1

**while**$\mathrm{fecg}\left(\text{idx\_QRS\_Peak}(i)-j\right)\leq \mathrm{fecg}\left(\text{idx\_QRS\_Peak}(i)-j+1\right)$**do**

j←j+1

end while

**while**$\mathrm{fecg}\left(\text{idx\_QRS\_Peak}(i)-j\right)>\mathrm{fecg}\left(\text{idx\_QRS\_Peak}(i)-j+1\right)$**do**

j←j+1

end while

idx_QRS_Onset(*i*) idx_QRS_Peak(*i*)−*j*

j←1

**while**$\mathrm{fecg}\left(\text{idx\_QRS\_Peak}(i)+j-1\right)\geq \mathrm{fecg}\left(\text{idx\_QRS\_Peak}(i)+j\right)$**do**

j←j+1

end while

**while**$\mathrm{fecg}\left(\text{idx\_QRS\_Peak}(i)+j-1\right)<\mathrm{fecg}\left(\text{idx\_QRS\_Peak}(i)+j\right)$**do**

j←j+1

end while

idx_QRS_Offset(*i*) idx_QRS_Peak(*i*) + *j*

else

 · · ·{//When QRS complex protrudes downward, code is same with reversing directions of inequality signs.}

**end ifend for**

### Feature formation for classification problems

We deal with the flattened waveform, *fecg*(*n*), to obtain the values of the features. We take voltage level of QRS onset point as the reference from which we measure voltage deviation [2,28]. We denote the mean value of electric potentials at QRS onset points as $\bar{\mathrm{fecg}\left(\mathrm{QRS}\;\text{onset}\right)}$. We consider this value as an effective zero voltage, so we measure voltage deviation from the $\bar{\mathrm{fecg}\left(\mathrm{QRS}\;\text{onset}\right)}$.

**Figure 5:**
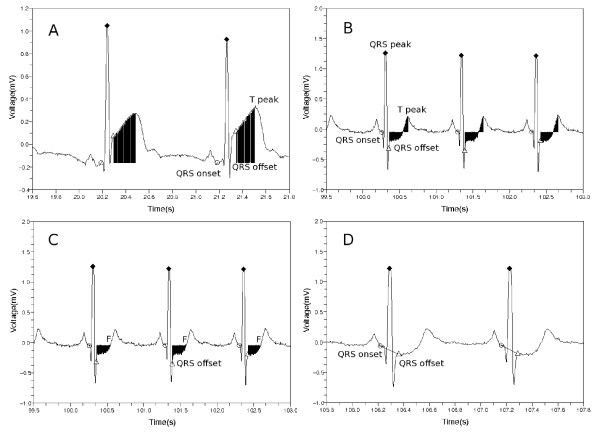
**Features used in the classification process.** Area between QRS offset and T peak with respect to the reference mean voltage $\bar{\mathrm{fecg}\left(\mathrm{QRS}\;\text{onset}\right)}$ in cases of (**a**) ST segment elevation and (**b**) ST segment depression. (**c**) Normalized and signed sum of voltage deviations from the QRS offset to the first point *F* at which voltage becomes equal to the reference voltage. (**d**) Slope from the QRS onset point to the QRS offset point. Markers ○,  and  designate QRS onset, peak and offset points respectively.

To form the first feature, we sum up all the voltage deviation from QRS offset point to T wave peak point as shown in Figure 5(a) and (b).

(2) $$\mathrm{featur}e_{1}=\overset{T\;\text{peak}}{\underset{i=\mathrm{QRS}\;\text{offset}}{\sum }}\left|\mathrm{fecg}(i)-\bar{\mathrm{fecg}\left(\mathrm{QRS}\;\text{onset}\right)}\right|$$

The second feature is similar to the first feature with an exception of the ending position of the sum. We terminate the summation as we reach the first point, F, at which the voltage becomes equal to the reference voltage $\bar{\mathrm{fecg}\left(\mathrm{QRS}\;\text{onset}\right)}$, see Figure 5. When doing this, we add the signed values of the voltage deviation to find whether the area is lower or higher with respect to the reference voltage. Then we divide the value by the voltage at QRS peak point. The second feature value is given as follows.

(3) $$\mathrm{featur}e_{2}=\left(\overset{F}{\underset{i=\mathrm{QRS}\;\text{offset}}{\sum }}\left(\mathrm{fecg}(i)-\bar{\mathrm{fecg}\left(\mathrm{QRS}\;\text{onset}\right)}\right)\right)/\left|\mathrm{fecg}\left(\mathrm{QRS}\;\text{peak}\right)\right|$$

The third feature is a slope from the QRS onset point to the QRS offset point.

(4) $$\mathrm{featur}e_{3}=\left|\frac{\mathrm{fecg}\left(\mathrm{QRS}\;\text{offset}\right)-\mathrm{fecg}\left(\mathrm{QRS}\;\text{onset}\right)}{\mathrm{QRS}\;\text{offset}-\mathrm{QRS}\;\text{onset}}\right|$$

We calculate these three feature values for each heart beat. Then we average these values in five successive beats, and arrange the three mean values as $\left(\mathrm{featur}e_{1},\mathrm{featur}e_{2},\mathrm{featur}e_{3}\right)$.

Algorithm 2 shows the pseudo-code of computing feature values.

#### 

##### Algorithm 2

A procedure to compute feature values. *nBeats*denotes the number of QRS peaks. It is the length of the sequences idx_QRS_Onset(*n*), idx_QRS_Peak(*n*) and idx_QRS_Offset(*n*). *n*_*cl*_ is equal to *nBeats*/5. **Input:***fecg*(*n*), idx_QRS_Onset(*n*), idx_QRS_Peak(*n*), idx_QRS_Offset(*n*)**Output:**$\left\{x_{1}^{\left(\mathrm{cl}\right)},x_{2}^{\left(\mathrm{cl}\right)},\cdot \cdot \cdot \;,x_{n_{\mathrm{cl}}}^{\left(\mathrm{cl}\right)}\right\}$ {//*cl* can be *S* (ST episode) or *N* (normal).} mean_idx_diff_1_$\left(\overset{\mathrm{nBeats}}{\underset{i=1}{\sum }}\left(\text{idx\_QRS\_Peak}(i)-\text{idx\_QRS\_Onset}(i)\right)\right)/\mathrm{nBeats}$mean_idx_diff_2_$\left(\overset{\mathrm{nBeats}}{\underset{i=1}{\sum }}\left(\text{idx\_QRS\_Offset}(i)-\text{idx\_QRS\_Peak}(i)\right)\right)/\mathrm{nBeats}$ {//mean_idx_diff_1_and mean_idx_diff_2_are truncated into integers.} $\bar{\mathrm{fecg}\left(\mathrm{QRS}\;\text{onset}\right)}$$\left(\overset{\mathrm{nBeats}}{\underset{i=1}{\sum }}\mathrm{fecg}\left(\text{idx\_QRS\_Onset}(i)\right)\right)/\mathrm{nBeats}$**for***i*=1 to *nBeats***do***k* idx_QRS_Peak(*i*) + mean_idx_diff_2_$\mathrm{featur}e_{1}(i)\leftarrow \overset{T\;\text{peak}}{\underset{j=k}{\sum }}\left|\mathrm{fecg}(j)-\bar{\mathrm{fecg}\left(\mathrm{QRS}\;\text{onset}\right)}\right|$$\mathrm{featur}e_{2}(i)\leftarrow \left(\overset{F}{\underset{j=k}{\sum }}\left(\mathrm{fecg}(j)-\bar{\mathrm{fecg}\left(\mathrm{QRS}\;\text{onset}\right)}\right)\right)/\left|\mathrm{fecg}\left(\text{idx\_QRS\_Peak}(i)\right)\right|$*m* idx_QRS_Peak(*i*)−mean_idx_diff_1_$\mathrm{featur}e_{3}(i)\leftarrow \left|\frac{\mathrm{fecg}(k)-\mathrm{fecg}(m)}{k-m}\right|$**end forfor***i*=1 to *nBeats*/5${\left[x_{i}^{\left(\mathrm{cl}\right)}\right]}_{1}\leftarrow \frac{1}{5}\overset{5i}{\underset{j=5i-4}{\sum }}\mathrm{featur}e_{1}(j)$${\left[x_{i}^{\left(\mathrm{cl}\right)}\right]}_{2}\leftarrow \frac{1}{5}\overset{5i}{\underset{j=5i-4}{\sum }}\mathrm{featur}e_{2}(j)$${\left[x_{i}^{\left(\mathrm{cl}\right)}\right]}_{3}\leftarrow \frac{1}{5}\overset{5i}{\underset{j=5i-4}{\sum }}\mathrm{featur}e_{3}(j)$**end for**

### Classification by kernel density estimation

We approximate probability density at a point by considering the other points. Let us assume we have *d*-dimensional points $\left\{x_{1},x_{2},\cdot \cdot \cdot \;,x_{n}\right\}$. We can estimate the probability density at a point **y** as $p\left(y\right)=\frac{1}{n}\frac{K}{V}$ where *V * is a small volume around **y**, and *K* is a number of enclosed points in the volume *V *[29]. We replace the term $\frac{K}{V}$ by *d*-dimensional Gaussian function as follows [30].

(5) $$p\left(y\right)=\frac{1}{n}\frac{K}{V}=\frac{1}{n}\overset{n}{\underset{i=1}{\sum }}\frac{1}{{\left(\sqrt{2\Pi }\right)}^{d}{\left|\sum \right|}^{1/2}}e^{-\frac{1}{2}{\left(y-x_{i}\right)}^{T}\overset{-1}{\sum }\left(y-x_{i}\right)}$$

If we assume that the covariance matrix  is a diagonal matrix with each diagonal element $b_{j}^{2}$$\left(1\leq j\leq d\right)$, the probability density at the point **y** is given as follows [31].

(6) $$p\left(y\right)=\frac{1}{n}\overset{n}{\underset{i=1}{\sum }}\frac{1}{{\left(\sqrt{2\Pi }\right)}^{d}\left(b_{1}b_{2}\cdot \cdot \cdot b_{d}\right)}e^{-\frac{1}{2}\overset{d}{\underset{j=1}{\sum }}{\left(\frac{{\left[y\right]}_{j}-{\left[x_{i}\right]}_{j}}{b_{j}}\right)}^{2}}$$

We classify a test point by examining posterior probabilities in which the test point belongs to two classes, normal or ischemic ST episode. We assume we have *n*_*S*_ points $\left\{x_{1}^{\left(S\right)},x_{2}^{\left(S\right)},\cdot \cdot \cdot \;,x_{n_{S}}^{\left(S\right)}\right\}$, and *n*_*N*_ points $\left\{x_{1}^{\left(N\right)},x_{2}^{\left(N\right)},\cdot \cdot \cdot \;,x_{n_{N}}^{\left(N\right)}\right\}$. The first and the second set designate training sets of ischemic ST episode and normal part, respectively. Each point is described by three components $\left(\mathrm{featur}e_{1},\mathrm{featur}e_{2},\mathrm{featur}e_{3}\right)$.

We compute posterior probability in which the test point **y**belongs to each class by Bayes’ theorem as follows [29].

(7) $$P\left(\text{class}|y\right)=\frac{P\left(\text{class}\right)p\left(y|\text{class}\right)}{P\left(\text{class}=N\right)p\left(y|\text{class}=N\right)+P\left(\text{class}=S\right)p\left(y|\text{class}=S\right)}$$

The prior probability $P\left(\text{class}\right)$ is given as $P\left(\text{class}=N\right)=n_{N}/\left(n_{N}+n_{S}\right)$ or $P\left(\text{class}=S\right)=n_{S}/\left(n_{N}+n_{S}\right)$. The likelihood $p\left(y|\text{class}=N\right)$ and $p\left(y|\text{class}=S\right)$ reads as

(8) $$p\left(y|\text{class}=N\right)=\frac{1}{n_{N}{\left(\sqrt{2\Pi }\right)}^{3}\left(b_{1}^{\left(N\right)}b_{2}^{\left(N\right)}b_{3}^{\left(N\right)}\right)}\overset{n_{N}}{\underset{i=1}{\sum }}e^{-\frac{1}{2}\overset{3}{\underset{j=1}{\sum }}{\left(\frac{{\left[y\right]}_{j}-{\left[x_{i}^{\left(N\right)}\right]}_{j}}{b_{j}^{\left(N\right)}}\right)}^{2}},$$

(9) $$p\left(y|\text{class}=S\right)=\frac{1}{n_{S}{\left(\sqrt{2\Pi }\right)}^{3}\left(b_{1}^{\left(S\right)}b_{2}^{\left(S\right)}b_{3}^{\left(S\right)}\right)}\overset{n_{S}}{\underset{i=1}{\sum }}e^{-\frac{1}{2}\overset{3}{\underset{j=1}{\sum }}{\left(\frac{{\left[y\right]}_{j}-{\left[x_{i}^{\left(S\right)}\right]}_{j}}{b_{j}^{\left(S\right)}}\right)}^{2}}.$$

The quantities $b_{i}^{\left(N\right)}$ and $b_{i}^{\left(S\right)}$$\left(1\leq i\leq 3\right)$ are called kernel bandwidths. We calculate these bandwidths for each class (N or S) and component $\left(1\leq i\leq 3\right)$. These kernel bandwidths impact accuracy of kernel density estimation [32].

We have *n*_*cl*_training points $\left\{x_{1}^{\left(\mathrm{cl}\right)},x_{2}^{\left(\mathrm{cl}\right)},\cdot \cdot \cdot \;,x_{n_{\mathrm{cl}}}^{\left(\mathrm{cl}\right)}\right\}$ where *cl* denotes class, *N* (normal) or *S* (ischemic ST episode). For each component $\left(1\leq i\leq 3\right)$ of the feature vector, we calculate the mean value of differences as follows.

(10) $$\mathrm{mea}n_{i}^{\left(\mathrm{cl}\right)}=\frac{1}{n_{\mathrm{cl}}\left(n_{\mathrm{cl}}-1\right)/2}\overset{n_{\mathrm{cl}}}{\underset{j=1}{\sum }}\overset{n_{\mathrm{cl}}}{\underset{k=j+1}{\sum }}\left|{\left[x_{j}^{\left(\mathrm{cl}\right)}\right]}_{i}-{\left[x_{k}^{\left(\mathrm{cl}\right)}\right]}_{i}\right|$$

We choose half of the mean, $\frac{1}{2}\mathrm{mea}n_{i}^{\left(\mathrm{cl}\right)}$, as kernel bandwidth $b_{i}^{\left(\mathrm{cl}\right)}$ for each class *cl* (*N* or *S*), and component *i*$\left(1\leq i\leq 3\right)$.

### Classification with the use of support vector machine

Let us assume we have *n*_*cl*_training points $\left\{x_{1}^{\left(\mathrm{cl}\right)},x_{2}^{\left(\mathrm{cl}\right)},\cdot \cdot \cdot \;,x_{n_{\mathrm{cl}}}^{\left(\mathrm{cl}\right)}\right\}$. Each point is described as $\left(\mathrm{featur}e_{1},\mathrm{featur}e_{2},\mathrm{featur}e_{3}\right)$ in a three-dimensional feature space. We construct support vector machine classifier by solving the following optimization problem [33]

(11) $$\begin{array}{cc} \underset{w,b,\xi }{\min} & \;\;\;\left\{\frac{1}{2}w^{T}\cdot w+C\overset{n_{\mathrm{cl}}}{\underset{j=1}{\sum }}\xi_{j}\right\}\\ \text{subject to} & \;\;\;t_{i}^{\left(\mathrm{cl}\right)}\left(w^{T}\cdot \phi \left(x_{i}^{\left(\mathrm{cl}\right)}\right)+b\right)\geq 1-\xi_{i},\;\;\;\xi_{i}\geq 0. \end{array}$$

The target label $t_{i}^{\left(\mathrm{cl}\right)}$ is specified as 1 (normal) or -1 (ischemic ST episode). The parameter *C* controls the trade-off between the slack variable (*ξ*_*i*_) penalty and the margin (**w**^*T*^·**w**) penalties [29]. The dual form of the above classifier reads as follows

(12) $$\begin{array}{cc} \underset{\alpha }{\max} & \;\left\{\overset{n_{\mathrm{cl}}}{\underset{j=1}{\sum }}\alpha_{j}-\frac{1}{2}\alpha^{T}\cdot H\alpha \right\}\\ \text{subject to} & \;\;\;\overset{n_{\mathrm{cl}}}{\underset{j=1}{\sum }}t_{j}^{\left(\mathrm{cl}\right)}\alpha_{j}=0,\;0\leq \alpha_{j}\leq C \end{array}$$

where the matrix *H* is expressed as $H_{\mathrm{ij}}\equiv t_{i}^{\left(\mathrm{cl}\right)}t_{j}^{\left(\mathrm{cl}\right)}K\left(x_{i}^{\left(\mathrm{cl}\right)},x_{j}^{\left(\mathrm{cl}\right)}\right)=t_{i}^{\left(\mathrm{cl}\right)}t_{j}^{\left(\mathrm{cl}\right)}\phi \left(x_{i}^{\left(\mathrm{cl}\right)}\right)\cdot \phi \left(x_{j}^{\left(\mathrm{cl}\right)}\right)=t_{i}^{\left(\mathrm{cl}\right)}t_{j}^{\left(\mathrm{cl}\right)}e^{-\frac{1}{3}{∥x_{i}^{\left(\mathrm{cl}\right)}-x_{j}^{\left(\mathrm{cl}\right)}∥}^{2}}$[33]. When we classify a new pattern **y**, we examine decision function, $\mathrm{sgn}\left(\overset{n_{\mathrm{cl}}}{\underset{j=1}{\sum }}t_{j}^{\left(\mathrm{cl}\right)}\alpha_{j}K\left(x_{j}^{\left(\mathrm{cl}\right)},y\right)+b\right)$. Whenever the input training set $\left\{x_{1}^{\left(\mathrm{cl}\right)},x_{2}^{\left(\mathrm{cl}\right)},\cdot \cdot \cdot \;,x_{n_{\mathrm{cl}}}^{\left(\mathrm{cl}\right)}\right\}$ was changed, we varied the parameter *C* to find its value which produced the highest classification rate.

### Experiments setting

We used kernel density estimation and support vector machine methods to evaluate the proposed approach. We completed the experiment for each channel and record available in the European ST-T database. First, we trained the classifier based on a subset of ST episodes and normal ECG. Then we tested how well the feature values discriminated the two classes, ST episode and normal. When we formed the ST episode data, we used all the ischemic ST episodes except ST deviations data resulted from non-ischemic causes such as position related changes in the electrical axis of the heart. To preserve balance between ST episode and normal ECG data, we collected normal data from the beginning of each record as much as the amount of ST episode data. When dividing the data into training and test sets, we assigned one tenth of data to the training data, and the rest to the test data. In the cases of e0106 lead 0, e0110, e0136, e0170, e0304, e0601, and e0615 records, we constructed the training data of one third of all data and test data of two thirds because these records had much small ischemic ST episode data. To avoid ambiguous region between ischemic ST episode and normal ECG, we removed 10 seconds amount of ECG data from each side of the boundary.

When we classified a test set $\left\{y_{i}\right\}$, four quantities were computed: true positive (TP), false negative (FN), false positive (FP), and true negative (TN). TP is a number of ischemic events correctly detected. FN is a number of erroneously rejected (missed) ischemic events. FP is a number of non-ischemic, that is, normal parts which the classifier erroneously detected as ischemic events. TN is a number of normal parts which our classifier correctly rejected as non-ischemic events [34]. These are numbers of corresponding **y**_*i*_ points which were obtained by averaging three feature values of successive five beats in Algorithm 2. The sensitivity and specificity are expressed in a usual fashion, *Se*=*TP*/(*TP* + *FN*) and *Sp*=*TN*/(*TN* + *FP*) respectively [6].

We tested the classifiers by counting how many ST episodes were correctly caught, out of 367 episodes in the 85 records of European ST-T database. For an interval of ischemic ST episode data, we formed *n* test points $\left\{y_{1},y_{2},\cdot \cdot \cdot \;,y_{n}\right\}$ from the data (Algorithm 2), and classified each test point and then counted numbers of two classes, “ischemic” and “normal”. If the number of class “ischemic” was larger than *n*/2, we declared the interval to be an ischemic ST episode. The experiments were completed for 367 ischemic ST episodes.

We compared the results of kernel density estimation (KDE) and support vector machine (SVM) methods with those formed by artificial neural network (ANN). The corresponding ANN classifier exhibits the following topology. The input layer has three nodes which accept *feature*_1_*feature*_2_ and *feature*_3_respectively. The output layer has two nodes which have target values $\left(1,0\right)$ and $\left(0,1\right)$ in the cases of “ischemia” and “normal” classes, respectively. We initialized bias weights as 0, and assigned random values between -1.0 and 1.0 to the weights of the network. The learning was carried out by running the backpropagation method [17] for 3000 iterations. We used a sigmoid activation function $1/\left(1+e^{-x}\right)$ and set learning rate 0.01. We adopted various topologies of hidden layers such as 3→(5)→(5)→23→(6)→23→(7)→2 and 3→(8)→2 where the number in each parenthesis represents a number of nodes in the corresponding hidden layer. We used stochastic (incremental) gradient descent method to alleviate some drawbacks of the standard gradient descent method, see [17].

## Results

### KDE with various kernels

We can use various kernels in kernel density estimation. If we have training points $\left\{x_{1},x_{2},\cdot \cdot \cdot \;,x_{n}\right\}$ and a test point **y**, the probability density at **y** is given as follows [35].

(13) $$p\left(y\right)=\frac{1}{n}\overset{n}{\underset{i=1}{\sum }}\frac{k_{G}}{b_{1}b_{2}b_{3}}e^{-\frac{1}{2}u_{i}^{2}}\;(\text{Gaussian}),$$

(14) $$p\left(y\right)=\frac{1}{n}\overset{n}{\underset{i=1}{\sum }}\frac{k_{R}}{b_{1}b_{2}b_{3}}1_{\left\{\left|u_{i}\right|\leq 1\right\}}\;(\text{Rectangular}),$$

(15) $$p\left(y\right)=\frac{1}{n}\overset{n}{\underset{i=1}{\sum }}\frac{k_{E}}{b_{1}b_{2}b_{3}}\left(1-u_{i}^{2}\right)1_{\left\{\left|u_{i}\right|\leq 1\right\}}\;(\text{Bartlett-Epanechnikov}),$$

(16) $$p\left(y\right)=\frac{1}{n}\overset{n}{\underset{i=1}{\sum }}\frac{k_{B}}{b_{1}b_{2}b_{3}}{\left(1-u_{i}^{2}\right)}^{2}1_{\left\{\left|u_{i}\right|\leq 1\right\}}\;(\text{Byweight}),$$

(17) $$p\left(y\right)=\frac{1}{n}\overset{n}{\underset{i=1}{\sum }}\frac{k_{\mathrm{Triw}}}{b_{1}b_{2}b_{3}}{\left(1-u_{i}^{2}\right)}^{3}1_{\left\{\left|u_{i}\right|\leq 1\right\}}\;(\text{Triweight}),$$

(18) $$p\left(y\right)=\frac{1}{n}\overset{n}{\underset{i=1}{\sum }}\frac{k_{\mathrm{Tria}}}{b_{1}b_{2}b_{3}}\left(1-u_{i}\right)1_{\left\{\left|u_{i}\right|\leq 1\right\}}\;(\text{Triangular}).$$

Here *k*_*G*_, *k*_*R*_, *k*_*E*_, *k*_*B*_, *k*_*Triw*_ and *k*_*Tria*_ are constants, and $u_{i}^{2}$ is given as $u_{i}^{2}\equiv \overset{3}{\underset{j=1}{\sum }}{\left(\frac{{\left[y\right]}_{j}-{\left[x_{i}\right]}_{j}}{b_{j}}\right)}^{2}$ because we use three feature values. The indicator function $1_{\left\{\left|u_{i}\right|\leq 1\right\}}$ is given as follows.

(19) $$1_{\left\{\left|u_{i}\right|\leq 1\right\}}=\left\{\begin{array}{ll} 1 & \left(\mathrm{if}\;\left|u_{i}\right|\leq 1\right)\\ 0 & \left(\text{otherwise}\right) \end{array}\right)$$

Table 1 shows classification results for various kernels. In all cases we used Daubechies8 wavelet to produce training and test sets. We took each bandwidth $b_{i}^{\left(\mathrm{cl}\right)}={\mathrm{mean}}_{i}^{\left(\mathrm{cl}\right)}\cdot \mathrm{factor}$ for class *cl*, ischemic or normal, and 1≤*i*≤3. The “detect” means how many ST episodes our classifier correctly detected, out of total 367 episodes. The “factor” in this table specifies how we multiplied on the ${\mathrm{mean}}_{i}^{\left(\mathrm{cl}\right)}$ to form the kernel bandwidth $b_{i}^{\left(\mathrm{cl}\right)}$. We varied this factor from 0.1 to 3.0, and selected the one for which a sum of sensitivity and specificity values attains a maximum. Because the Gaussian kernel produced best results, in the sequel we will use the Gaussian kernel. Table 2 shows the results with respect to various kernel bandwidths.

**Table 1:** Classification results with respect to various kernels


**Kernel**	**Factor**	**Se.**	**Sp.**	**TP**	**TN**	**FP**	**FN**	**Detect**
Gaussian	0.5	0.939	0.912	27600	21441	2075	1794	349
Rectangular	1.5	0.892	0.913	26209	21460	2056	3185	329
Epanechnikov	1.7	0.904	0.915	26583	21522	1994	2811	335
Byweight	2.0	0.912	0.916	26794	21533	1983	2600	333
Triweight	2.1	0.916	0.916	26923	21542	1974	2471	336
Triangular	1.8	0.908	0.917	26680	21554	1962	2714	334

**Table 2:** Classification results of Gaussian kernels with respect to various bandwidths


**Factor**	**Se.**	**Sp.**	**TP**	**TN**	**FP**	**FN**	**Detect**
0.2	0.943	0.867	27728	20399	3117	1666	353
0.3	0.944	0.893	27745	20996	2520	1649	352
0.4	0.942	0.905	27697	21279	2237	1697	351
0.5	0.939	0.912	27600	21441	2075	1794	349
0.6	0.934	0.915	27453	21526	1990	1941	343
0.7	0.929	0.916	27318	21550	1966	2076	338
0.8	0.924	0.916	27148	21529	1987	2246	337

### Results for KDE, SVM and ANN with various wavelets

**Figure 6:**
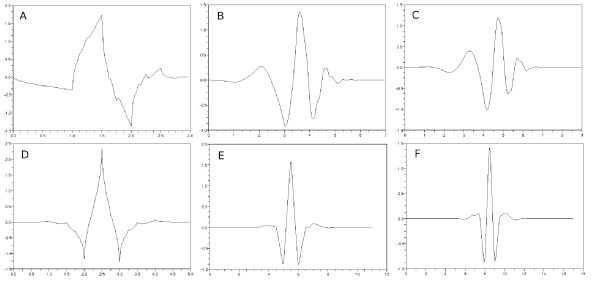
**Shapes of various wavelets.** (**a**) Daubechies4, (**b**) Daubechies8, (**c**) Daubechies10, (**d**) Coiflet6, (**e**) Coiflet12 and (**f**) Coiflet18.

We examined the classifiers to find out how their performance depends on the mother wavelets which were used to produce training and test sets in Algorithm 1. We used 7 wavelets, Haar, Daubechies4, Daubechies8, Daubechies10, Coiflet6, Coiflet12 and Coiflet18 [22,36]. The number forming a part of the name of each wavelet designates the length of filter which characterizes corresponding wavelet. Figure 6 shows selected shapes of wavelet functions except for the Haar wavelet which is given as

(20) $$\mathrm{Haar}(t)=\left\{\begin{array}{c} 1\;\;\left(0\leq t\leq 1/2\right)\\ -1\left(1/2\leq t\leq 1\right) \end{array}\right).$$

Table 3 shows the classification results obtained for KDE. The kernel bandwidth is expressed as $b_{i}^{\left(\mathrm{cl}\right)}={\mathrm{mean}}_{i}^{\left(\mathrm{cl}\right)}/2$ for each class *cl* and 1≤*i*≤3. We used Gaussian kernel.

**Table 3:** Classification results for KDE with respect to various wavelets


**Wavelet**	**Se.**	**Sp.**	**TP**	**TN**	**FP**	**FN**	**Detect**
Haar	0.915	0.893	25906	20245	2420	2418	339
Daubechies4	0.936	0.906	27488	21130	2186	1886	343
Daubechies8	0.939	0.912	27600	21441	2075	1794	349
Daubechies10	0.942	0.916	27862	21585	1969	1710	348
Coiflet6	0.934	0.900	28586	21837	2430	2027	349
Coiflet12	0.932	0.914	27846	21757	2041	2045	349
Coiflet18	0.937	0.919	27721	21612	1911	1859	349

Table 4 shows the classification results for the KDE with respect to various bandwidths and wavelets. The first column for each wavelet item represents the sum of sensitivity and specificity. The second column shows how many ST episodes were correctly detected. We used the kernel bandwidths $b_{i}^{\left(\mathrm{cl}\right)}={\mathrm{mean}}_{i}^{\left(\mathrm{cl}\right)}\cdot \mathrm{factor}$ for each class *cl* and 1≤*i*≤3. The sum of sensitivity and specificity becomes maximum when the bandwidth $b_{i}^{\left(\mathrm{cl}\right)}$ is around $b_{i}^{\left(\mathrm{cl}\right)}\approx {\mathrm{mean}}_{i}^{\left(\mathrm{cl}\right)}/2$.

**Table 4:** Classification results for KDE versus selected values of bandwidths and types of wavelets


**Factor**	**Haar**		**Daub4**		**Daub8**		**Daub10**		**Coif6**		**Coif12**		**Coif18**	
0.2	1.770	348	1.797	348	1.811	353	1.817	355	1.794	356	1.812	360	1.825	354
0.3	1.797	350	1.825	350	1.837	352	1.843	356	1.822	354	1.833	357	1.848	353
0.4	1.808	345	1.838	344	1.847	351	1.856	353	1.833	354	1.844	355	1.857	354
0.5	1.808	339	1.842	343	1.851	349	1.859	348	1.834	349	1.846	349	1.856	349
0.6	1.806	336	1.840	339	1.849	343	1.857	343	1.832	345	1.843	346	1.854	346
0.7	1.800	331	1.836	336	1.846	338	1.853	338	1.829	340	1.837	341	1.849	344
0.8	1.792	325	1.829	334	1.839	337	1.848	335	1.824	334	1.831	339	1.842	340

Table 5 shows the classification results obtained for SVM. The parameter *C* controls the trade-off between the slack variable (*ξ*_*i*_) penalty and the margin (**w**^*T*^·**w**) penalties. We examined the classification accuracy versus the values of *C* changing from 0.1 to 300.0 in step of 0.1, and selected the one that made the sum of sensitivity and specificity maximal.

**Table 5:** Classification results for SVM for various wavelets


**Wavelet**	***C***	**Se.**	**Sp.**	**TP**	**TN**	**FP**	**FN**	**Detect**
Haar	291.3	0.924	0.907	26163	20547	2118	2161	345
Daubechies4	242.9	0.937	0.923	27527	21519	1797	1847	349
Daubechies8	245.5	0.941	0.923	27658	21712	1804	1736	355
Daubechies10	174.3	0.943	0.927	27894	21838	1716	1678	349
Coiflet6	288.2	0.933	0.918	28571	22284	1983	2042	348
Coiflet12	52.8	0.929	0.918	27757	21858	1940	2134	348
Coiflet18	23.4	0.936	0.927	27692	21805	1718	1888	352

Table 6 shows the classification results obtained by ANN. The number in parenthesis represents the number of nodes in the corresponding hidden layer. The first, second and third column express sensitivity, specificity and the “detect” respectively. We experimented 10 times, and averaged the results because we obtained different results each time due to the random initialization of weights.

**Table 6:** Results for ANN classifiers with respect to various wavelets and sizes of hidden layers


**Wavelet**	3→(5)→(5)→2			3→(6)→2			3→(7)→2			3→(8)→2		
Haar	0.851	0.920	311.8	0.866	0.916	319.2	0.867	0.917	319.1	0.867	0.916	320.7
Daub4	0.866	0.932	304.2	0.881	0.930	313.7	0.880	0.932	315.2	0.881	0.931	317.8
Daub8	0.864	0.931	307.2	0.878	0.929	317.9	0.875	0.931	319.4	0.880	0.932	319.4
Daub10	0.866	0.939	312.5	0.882	0.935	321.8	0.885	0.935	325.5	0.882	0.937	325.1
Coif6	0.848	0.930	311.2	0.868	0.920	319.3	0.863	0.923	319	0.872	0.927	321
Coif12	0.855	0.936	310.5	0.868	0.933	318.2	0.866	0.935	319.6	0.874	0.935	319.8
Coif18	0.874	0.938	311.8	0.883	0.937	319.2	0.884	0.936	320.2	0.886	0.937	323.5

Tables 3,5 and 6 show that the Daubechies8 and Daubechies10 wavelets give us superior results. The shapes of these two wavelets are similar to typical ECG waveforms [37,38]. From now on, we use the Daubechies8 wavelet exclusively.

### Effects of baseline wandering in ECG

Table 7 shows the classification results by KDE, SVM and ANN when we did not remove baseline wandering in ECG. If we compare this table with the Tables 3, 5 and 6, we get to know it is essential to remove baseline wandering in Algorithm 1. In the Table 7, we selected the kernel bandwidths $b_{i}^{\left(\mathrm{cl}\right)}$ in the KDE classifier as $b_{i}^{\left(\mathrm{cl}\right)}={\mathrm{mean}}_{i}^{\left(\mathrm{cl}\right)}/2$, $\left(1\leq i\leq 3\right)$. We used the ANN classifier with sizes of layers expressed as 3→(7)→2. The results of ANN were obtained by averaging results for 10 repetition of the experiments. The parameter *C* of the SVM classifier was 297.9.

**Table 7:** Classification results for KDE, SVM and ANN without removal of baseline wandering


	**Se.**	**Sp.**	**TP**	**TN**	**FP**	**FN**	**Detect**
KDE	0.852	0.837	22132	17211	3342	3839	328
SVM	0.870	0.835	22605	17161	3392	3366	326
ANN	0.785	0.827	20388.8	17005.7	3547.3	5582.2	296.1

If we use unsuitable wavelet scale like the one in Figure 3 to remove baseline wandering, it becomes difficult to obtain good results. As the sampling frequency was 250 Hz, we selected the wavelet scale $\left\lceil \underset{2}{\log}250\right\rceil =8$ in Algorithm 1. Table 8 shows the classification results when wrong wavelet scales were selected. The kernel bandwidth setting in KDE and layer composition of ANN classifier were same as the Table 7. The middle row of wavelet scale 8 in Table 8 was our choice in Algorithm 1. Each entry in the row of wavelet scale 8 has counterparts in “Daubechies8” rows in Tables 3, 5 and 6.

**Table 8:** Classification results for KDE, SVM and ANN with incorrectly selected wavelet scales to remove baseline wandering


	**KDE**			**SVM**				**ANN**		
	**Se.**	**Sp.**	**Detect**	**Trade-off**	**Se.**	**Sp.**	**Detect**	**Se.**	**Sp.**	**Detect**
scale 6	0.851	0.785	319	288.2	0.842	0.818	318	0.748	0.864	277.5
scale 7	0.931	0.905	344	232.8	0.932	0.915	349	0.876	0.933	320.7
scale 8	0.939	0.912	349	245.5	0.941	0.923	355	0.875	0.931	319.4
scale 9	0.929	0.906	347	110.4	0.930	0.918	350	0.859	0.918	320.7
scale 10	0.921	0.896	340	97.5	0.916	0.907	343	0.838	0.896	313.2

### Effects of simulated noise

We examined performance of the classifiers when we added simulated noise into the original ECG signal. We modeled the noise as the sum of wandering baseline and AC power line 60 Hz noise.

Let us assume we have original signal data, *ecg*(*i*)$\left(1\leq i\leq n\right)$. First, we compute mean value and standard deviation of the ECG signal as $m=\left(\overset{n}{\underset{i=1}{\sum }}\mathrm{ecg}(i)\right)/n$ and $s=\sqrt{\left(\overset{n}{\underset{i=1}{\sum }}{\left(\mathrm{ecg}(i)\right)}^{2}\right)/n-m^{2}}$. Then we form a new signal *ecg*^*″*^(*i*) by

(21) $$\mathrm{ec}g^{\prime \prime }(i)=\mathrm{ecg}(i)+s\cdot a\cdot \left(\sin\left(b\cdot \frac{i}{\mathrm{Samp}\text{\_}\mathrm{Freq}}\right)+\frac{1}{2}\cos\left(2\Pi 60\cdot \frac{i}{\mathrm{Samp}\text{\_}\mathrm{Freq}}\right)\right)$$

where *a* is an amplification factor and *b* is an angular frequency of the added baseline. Here *Samp*_*Freq* means sampling frequency which was 250 Hz in our case. We varied *a* from 0.1 to 1.0 in step of 0.1, and selected *b* to be equal to 2, 4 or 6.

**Figure 7:**
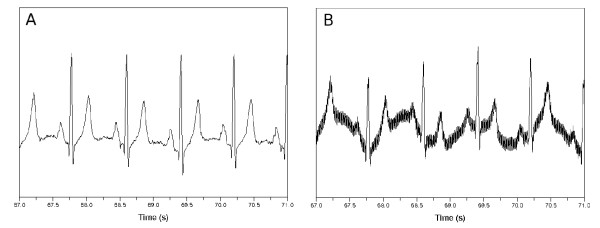
**ECG signal affected by synthetic noise.** (**a**) Original signal. (**b**) Noise-affected signal when *a* is 1.0 and *b* is 6.0.

Figure 7 shows the original ECG and its noise-impacted version. Tables 9, 10 and 11 show the experimental results for the noisy ECG signal. The first, second and third column in each *b* item represent the sensitivity, specificity and the “detect” respectively. The kernel bandwidth is set as $b_{i}^{\left(\mathrm{cl}\right)}={\mathrm{mean}}_{i}^{\left(\mathrm{cl}\right)}/2$ for the KDE classifier. The layer composition of the ANN classifier was 3→(7)→2. The first column in each *b* item in Table 10 includes the trade-off parameter *C* which produced best results.

**Table 9:** Classification results for KDE versus varying intensity of noise


**a**	**b=2**			**b=4**			**b=6**		
0.1	0.937	0.903	346	0.934	0.902	346	0.933	0.904	346
0.2	0.933	0.893	346	0.927	0.892	341	0.916	0.879	337
0.3	0.929	0.884	342	0.915	0.873	340	0.908	0.856	338
0.4	0.925	0.864	346	0.903	0.852	344	0.885	0.834	327
0.5	0.916	0.858	346	0.882	0.848	333	0.871	0.817	333
0.6	0.906	0.866	343	0.872	0.832	329	0.858	0.803	321
0.7	0.891	0.856	340	0.859	0.815	325	0.848	0.794	322
0.8	0.887	0.850	339	0.852	0.799	325	0.838	0.793	317
0.9	0.881	0.849	341	0.837	0.798	312	0.837	0.778	301
1.0	0.870	0.844	335	0.832	0.787	317	0.824	0.779	319

**Table 10:** Classification results for SVM with varying intensity of the simulated noise


**a**	**b=2**				**b=4**				**b=6**			
0.1	79.0	0.936	0.920	350	254.6	0.940	0.915	352	147.4	0.936	0.916	349
0.2	143.0	0.929	0.914	347	168.5	0.929	0.904	349	33.9	0.922	0.888	339
0.3	84.1	0.923	0.910	348	50.9	0.915	0.891	341	72.3	0.902	0.875	337
0.4	124.5	0.919	0.899	348	51.3	0.903	0.869	341	52.0	0.888	0.848	330
0.5	65.1	0.909	0.895	347	86.7	0.882	0.859	333	79.4	0.872	0.828	328
0.6	96.7	0.903	0.887	343	67.3	0.879	0.844	330	71.6	0.865	0.809	323
0.7	119.3	0.888	0.880	339	27.2	0.868	0.832	329	36.6	0.856	0.796	313
0.8	201.8	0.893	0.863	334	24.9	0.851	0.829	320	27.1	0.848	0.785	310
0.9	278.2	0.888	0.857	335	16.7	0.841	0.816	308	71.0	0.850	0.777	315
1.0	211.7	0.879	0.859	324	58.2	0.835	0.806	311	20.3	0.837	0.776	317

**Table 11:** Classification results for ANN with varying intensity of the simulated noise


**a**	**b=2**			**b=4**			**b=6**		
0.1	0.880	0.929	322.2	0.870	0.921	320.8	0.874	0.922	320.3
0.2	0.867	0.928	322.8	0.844	0.922	315.1	0.840	0.905	314.2
0.3	0.863	0.929	319.3	0.837	0.906	313.4	0.811	0.895	303.4
0.4	0.856	0.917	320.5	0.823	0.887	312.3	0.784	0.874	300.6
0.5	0.830	0.918	320.1	0.811	0.874	309.1	0.778	0.850	300.3
0.6	0.818	0.916	314.9	0.803	0.858	299.2	0.762	0.833	292
0.7	0.803	0.910	309.6	0.759	0.865	281.9	0.761	0.811	283.2
0.8	0.814	0.898	308.6	0.753	0.839	288.2	0.745	0.801	280.7
0.9	0.798	0.892	303.4	0.723	0.838	273.9	0.728	0.795	261.7
1.0	0.777	0.890	297.1	0.724	0.807	265.3	0.728	0.788	269.3

### Comparison with others’ works

To compare our approach with others’ works, we tested the classifiers on 10 selected records, e0103, e0104, e0105, e0108, e0113, e0114, e0147, e0159, e0162 and e0206. Table 12 shows results of comparison. The papers by Papaloukas et al. [10], Goletsis et al. [39], Exarchos et al. [13] and Murugan et al. [15] in Table 12 dealt with the 10 records.

**Table 12:** Results of comparative analysis


**Researcher**	**Sensitivity**	**Specificity**
Papaloukas et al. [10]	0.90	0.90
Goletsis et al. [39]	0.912	0.909
Exarchos et al. [13]	0.912	0.922
Murugan et al. [15]	0.923	0.943
Present work by KDE	0.945	0.943
Present work by SVM	0.957	0.953

We used the Daubechies8 wavelet in Algorithm 1 to analyze the ECG waveform, and took the kernel bandwidths $b_{i}^{\left(\mathrm{cl}\right)}={\mathrm{mean}}_{i}^{\left(\mathrm{cl}\right)}/2$ for the KDE classifier with Gaussian kernel. We used the SVM classifier with *C*=281.1.

## Discussion

Table 1 showed how the classification results were dependent on various kernel functions in kernel density estimation. Gaussian kernel produced best results.

Tables 3, 4, 5 and 6 show how the classification results depend on mother wavelets used in Algorithm 1. Daubechies8 and Daubechies10 wavelets were best. Because we implemented wavelet transform program with the use of matrix multiplication, we selected Daubechies8 wavelet to reduce computational burden. Daubechies10 wavelet did not produce much better classification accuracy than Daubechies8 wavelet.

Tables 2 and 4 indicate that the choice of kernel bandwidths was reasonable. When we took the kernel bandwidths $b_{i}^{\left(\mathrm{cl}\right)}={\mathrm{mean}}_{i}^{\left(\mathrm{cl}\right)}/2$ for class *cl*, 1≤*i*≤3, we obtained best results except for the case of Coiflet18 wavelet. Even in the case, the best parameter $b_{i}^{\left(\mathrm{cl}\right)}=0.4\cdot {\mathrm{mean}}_{i}^{\left(\mathrm{cl}\right)}$ was close enough to $b_{i}^{\left(\mathrm{cl}\right)}=\mathrm{mea}n_{i}^{\left(\mathrm{cl}\right)}/2$. We maintained this choice in Tables 7, 8 and 9. In this way, we could automatically select 6 kernel bandwidths, and this exempted us from choosing any numerical parameters.

The SVM classifiers in Tables 5, 7 and 8 produced better results than the KDE classifiers, but they required us to determine optimal value of the parameter *C*. Whenever we used different wavelets on the same data set in Table 5, we had to choose different trade-off parameter *C*. This was also the case in Table 8 where we intentionally selected incorrect wavelet scales to remove baseline wandering.

Order of magnitude of *feature*_3_was very different from *feature*_1_and *feature*_2_. When we produced the feature values using Daubechies8 wavelet in Algorithm 1, mean values of $\left|{\mathrm{feature}}_{1}\right|$, $\left|{\mathrm{feature}}_{2}\right|$ and $\left|{\mathrm{feature}}_{3}\right|$ were 7.327, 7.613 and 0.004, respectively. Thus we had to normalize the feature values to use them in classification. Even if the orders of magnitude of *feature*_1_, *feature*_2_ and *feature*_3_were very different, the equation of kernel density estimation included a term $\frac{1}{b_{1}^{\left(\mathrm{cl}\right)}b_{2}^{\left(\mathrm{cl}\right)}b_{3}^{\left(\mathrm{cl}\right)}}\underset{i}{\sum }e^{-\frac{1}{2}\overset{3}{\underset{j=1}{\sum }}{\left(\frac{{\left[y\right]}_{j}-{\left[x_{i}^{\left(\mathrm{cl}\right)}\right]}_{j}}{b_{j}^{\left(\mathrm{cl}\right)}}\right)}^{2}}$. Furthermore each operand in the sum comes in the form of $\frac{{\left[y\right]}_{j}-{\left[x_{i}^{\left(\mathrm{cl}\right)}\right]}_{j}}{b_{j}^{\left(\mathrm{cl}\right)}}$, normalization by kernel bandwidth. We thought these would be helpful to overcome the difference of order of magnitude between *feature*_1_, *feature*_2_ and *feature*_3_. This was a main driving force to adopt the kernel density estimation.

We implemented the KDE and ANN classifier in C language for ourselves. For SVM classifier, we used libsvm library [33]. We compiled the programs with gcc and g++ without using any SIMD (single instruction multiple data) math library. Total amount of ECG text files which we used in our analysis was 200.4 MB. This amount is just about voltage information not including time information. When we ran our programs to process the ECG text files in Pentium4 3.2 GHz CPU, it took 243.0 seconds until the procedures of removing baseline wandering and detecting time positions in Algorithm 1 were completed. This was when we used Daubechies8 wavelet. Feature extraction in Algorithm 2, required 0.6 seconds. It took 1.2 seconds for the KDE classifier to process all the files. The SVM classifier required 0.8 seconds for the same job. The ANN classifier with layer composition 3→(7)→2 required 94.7 seconds.

We compared our QRS detection algorithm with Hamilton and Tompkins’ algorithm [40] which was implemented in C language as an open source software [41]. We supplanted the portion from DWT of *fecg*(*n*) to making *idx*_*QRS*_*Peak*(*n*) in Algorithm 1, with the Hamilton and Tompkins’ program. When we ran the modified program to process the procedures of removing baseline wandering and detecting time positions, it took 59.5 seconds which was approximately four times faster than ours. However the KDE classifier produced the results of $\left(\text{sensitivity},\text{specificity},\text{detect}\right)$ being (0.904, 0.891, 329). These results are somewhat inferior compared to the results in Table 2.

## Conclusions

The ST segment deviation in ECG can be an indicator of myocardial ischemia. If we can predict an ischemic syndrome as early as possible, we will be able to prevent more severe heart disease such as myocardial infarction [8,9].

To detect ischemic ST episode, we adopted a method directly using morphological features of ECG waveforms. We did not use weight tuning methods such as artificial neural network or decision tree because we wanted to show explicitly which features of ECG waveforms were meaningful to detect ischemic ST episodes. In this regard, we calculated three feature values for each heart beat. They were area between QRS offset and T-peak points, normalized and signed sum from QRS offset to effective zero voltage point, and slope from QRS onset to offset point. After calculating these feature values for each heart beat, we averaged the values of successive five beats because we wanted to reduce outlier effects. The order of magnitude of the third feature value, the slope from QRS onset to offset point, was very different from the other two feature values. To take care of this problem, we considered classification method by kernel density estimation.

We described how we removed baseline wandering in ECG, and detected time positions of QRS complexes by the discrete wavelet transform. Since our classifier selects automatically kernel bandwidths in kernel density estimation, virtually it does not require any numerical parameter which operator should provide. In the tests, SVM with optimal parameters showed just a slightly better classification accuracy than the proposed method, but finding those parameters is a heavy burden compared with the proposed method. We can conclude that overall our proposed method is efficient enough and has more advantages than existing methods.

## Competing interests

The authors declare that they have no competing interests.

## Authors’ contributions

Mr. Park implemented the whole algorithm, and wrote the manuscript. Dr. Pedrycz proofread it and provided useful technical comments. Dr. Jeon designed the experiments and checked the validity of the proposed methods. All authors read and approved the final manuscript.
